# Impact of the introduction of pneumococcal conjugate vaccine on immunization coverage among infants

**DOI:** 10.1186/1471-2431-5-43

**Published:** 2005-11-28

**Authors:** Nancy D Lin, Ken Kleinman, K Arnold Chan, Xian-Jie Yu, Eric K France, Stanley Xu, Feifei Wei, John Mullooly, Jeanne Santoli, Tracy A Lieu, the Vaccine Safety Datalink Group

**Affiliations:** 1 Department of Ambulatory Care and Prevention, Harvard Pilgrim Health Care and Harvard Medical School, Boston, MA, USA; 2 Harvard School of Public Health, Boston, MA, USA; 3 Kaiser Permanente, Denver, Kaiser Permanente, Denver, CO, USA; 4 HealthPartners Research Foundation, Minneapolis, MN, USA; 5 Center for Health Research, Kaiser Permanente Northwest, Portland, OR, USA; 6 National Immunization Program, Centers for Disease Control and Prevention, Atlanta, GA

## Abstract

**Background:**

The introduction of pneumococcal conjugate vaccine (PCV) to the U.S. recommended childhood immunization schedule in the year 2000 added three injections to the number of vaccinations a child is expected to receive during the first year of life. Surveys have suggested that the addition of PCV has led some immunization providers to move other routine childhood vaccinations to later ages, which could increase the possibility of missing these vaccines. The purpose of this study was to evaluate whether introduction of PCV affected immunization coverage for recommended childhood vaccinations among 13-month olds in four large provider groups.

**Methods:**

In this retrospective cohort study, we analyzed computerized data on vaccinations for 33,319 children in four large provider groups before and after the introduction of PCV. The primary outcome was whether the child was up to date for all non-PCV recommended vaccinations at 13 months of age. Logistic regression was used to evaluate the association between PCV introduction and the primary outcome. The secondary outcome was the number of days spent underimmunized by 13 months. The association between PCV introduction and the secondary outcome was evaluated using a two-part modelling approach using logistic and negative binomial regression.

**Results:**

Overall, 93% of children were up-to-date at 13 months, and 70% received all non-PCV vaccinations without any delay. Among the entire study population, immunization coverage was maintained or slightly increased from the pre-PCV to post-PCV periods. After multivariate adjustment, children born after PCV entered routine use were less likely to be up-to-date at 13 months in one provider group (Group C: OR = 0.5; 95% CI: 0.3 – 0.8) and were less likely to have received all vaccine doses without any delay in two Groups (Group B: OR = 0.4, 95% CI: 0.3 – 0.6; Group C: OR = 0.5, 95% CI: 0.4 – 0.7). This represented 3% fewer children in Group C who were up-to-date and 14% (Group C) to 16% (Group B) fewer children who spent no time underimmunized at 13 months after PCV entered routine use compared to the pre-PCV baseline. Some disruptions in immunization delivery were also observed concurrent with temporary recommendations to suspend the birth dose of hepatitis B vaccine, preceding the introduction of PCV.

**Conclusion:**

These findings suggest that the introduction of PCV did not harm overall immunization coverage rates in populations with good access to primary care. However, we did observe some disruptions in the timely delivery of other vaccines coincident with the introduction of PCV and the suspension of the birth dose of hepatitis B vaccine. This study highlights the need for continued vigilance in coming years as the U.S. introduces new childhood vaccines and policies that may change the timing of existing vaccines.

## Background

The addition of pneumococcal conjugate vaccine (PCV) to the U.S. recommended childhood immunization schedule in the year 2000 added three injections to the number of shots a child is expected to receive during the first year of life. Whereas seven to ten injections were recommended during the first year of life prior to introduction of PCV, between ten and thirteen injections are now recommended, depending on use of combination vaccines. With the addition of pneumococcal vaccination, the youngest children may receive up to five injections at a single office visit[1].

Simultaneous administration of vaccines is recommended to facilitate early protection against vaccine-preventable disease[2]. At the same time, administration of multiple injections may create distress for children and parents, and many parents and providers have previously expressed concern regarding the administration of four vaccines at a single visit [3-5]. It is unclear how increased crowding of the childhood immunization schedule and safety concerns about multiple injections related to the introduction of PCV have affected immunization delivery. Two regional provider surveys suggested that physicians who administer PCV may delay other vaccinations,[4,6] although a different, national survey found that most physicians who adopted PCV in their practices would administer four or more injections at the 2-month visit[7]. The objective of this study was to evaluate whether the introduction of PCV affected immunization delivery in actual practice among large populations of children in several provider groups.

## Methods

### Study population

This study included children enrolled in four large provider groups: Harvard Vanguard Medical Associates (Boston, MA), HealthPartners (Minneapolis, MN), Kaiser Permanente of Colorado (Denver, CO), and Kaiser Permanente Northwest (Portland, OR). These sites participate in the Centers for Disease Control and Prevention Vaccine Safety Datalink Project, in which individual-level vaccination, demographic, and medical data are shared to facilitate vaccine safety and other vaccine-related epidemiologic research[8].

We studied infants who were born into one of the four provider groups between October 1996 and November 2000 and had received at least one polio vaccination, where receipt of polio vaccination was used as an indicator that a child received immunizations that were recorded by the provider group information systems (n = 86,561). To ensure that the most complete immunization information was available, the study population was additionally restricted to children continuously enrolled throughout their first year of life (n = 38,588). The study protocol was approved by the institutional review boards at the four participating sites and the Centers for Disease Control and Prevention.

### Definition of Post-PCV and Pre-PCV exposure cohorts

Each child was assigned to one of two birth cohorts based on the timing of their birth relative to the regulatory approval of PCV in February 2000[9]. Individuals who were born between October 1996 and January 2000 were assigned to the "pre-PCV" birth cohort. Children born between February and November 2000 were assigned to the "post-PCV" birth cohort.

While introduction of PCV added three new vaccine injections, use of the hepatitis B-*Haemophilus influenzae type B *(Hib) combination vaccine can offset the increase in vaccine injections a child requires to be fully immunized during the first year of life. Hepatitis B-Hib combination vaccine was available throughout the study period in one provider group and was implemented in two other provider groups during the study period; in the fourth provider group, it was not available at all. Table 1 describes how the expected number of injections varied based on provider group-specific availability of the hepatitis B-Hib combination vaccine during the pre-PCV and the post-PCV periods.

**Table 1:** Variation in the expected number of vaccine injections during the first year of life, by provider group and PCV policy period

	Number of vaccine injections expected during the first year of life	
Group	Pre-PCV birth cohort	Post-PCV birth cohort

Group A†	7* or 10	10* or 13
Group B‡	10	13
Group C§	10	10* or 13
Group D||	7* or 10	10* or 13

A child born during the pre-PCV period is expected to receive 3 DTP vaccinations, 2 polio vaccinations, 3 Hib vaccinations, and 2 hepatitis B vaccinations during the first year of life. Introduction of PCV added three new vaccine injections.

* Replacement of the separate hepatitis B and Hib vaccines during the first year of life with 2 doses of hepatitis B-Hib combination vaccine reduces the number of vaccine injections expected during the first year of life to 7 shots in the pre-PCV period and 10 in the post-PCV period.

† Hepatitis B-Hib combination vaccine available throughout the study period.

‡ Hepatitis B-Hib combination vaccine never available during the study period.

§ Hepatitis B-Hib combination vaccine available starting in 2000 following PCV introduction, based on descriptive analyses.

|| Hepatitis B-Hib combination vaccine available starting in mid-1999 prior to PCV introduction, based on descriptive analyses.

### Definition of immunization coverage measures

We assessed the impact of the introduction of PCV on two measures of immunization coverage at 13 months of age: (1) up-to-date status and (2) time spent underimmunized[10].

Vaccination histories were identified using the immunizations databases for each provider group. When vaccine entries of the same type were recorded within seven days of one another, the later entry was assumed to represent a duplicate record and was excluded (0.006% – 0.56%, by vaccine type). In addition, vaccinations that were administered before the minimum recommended age or earlier than the minimum recommended between-vaccination interval, allowing for a four-day grace period,[1,2] were considered to be invalid. Only the remaining vaccinations for eligible individuals were included in our analyses.

In general, a child was considered to be up-to-date for non-PCV recommended vaccinations at 13 months of age if they received all of the following: 3 diphtheria and tetanus toxoids and acellular or whole cell pertussis (DTP) vaccinations; 2 polio vaccinations; 2 hepatitis B vaccinations; and 3 Hib vaccinations. Children who received the hepatitis B-Hib combination vaccine were considered up-to-date for the hepatitis B and Hib vaccinations if they received 2 hepatitis B vaccine doses and 2 Hib vaccine doses by age 13 months.

The up-to-date measure includes only those vaccine doses with recommended age ranges contained wholly included within the 13-month individual follow-up period. Doses with recommended age ranges that spanned the 13-month birthday (e.g., third dose of hepatitis B vaccine recommended between 6 and 18 months of age) were not included because children who had not yet received these doses by age 13 months would not be considered late. As a result, the up-to-date definition corresponds to vaccinations recommended between birth and 6 months of age. PCV was not included in the outcome definition because the primary study objective was to evaluate whether addition of PCV affected adherence to existing vaccine recommendations.

The secondary outcome, time spent underimmunized, was defined as the number of days a child spent underimmunized for at least one non-PCV recommended vaccination by 13 months of age and is the complement of a previously documented outcome measure, cumulative time spent up-to-date[11]. Because it measures the amount of vaccination delay rather than immunization status at a single point in time, this outcome is expected to be more sensitive than up-to-date status at 13 months. Operationally, we calculated time spent underimmunized for each individual by assessing the child's up-to-date status for the non-PCV recommended vaccinations on each day from birth up to their 13-month birthday based on the U.S. recommended childhood immunization schedule[1]. We then summed the number of days on which the child was not up-to-date for at least one non-PCV recommended vaccination. When the recommended age range was specified in months, a vaccination was considered age-appropriate if it was given prior to the end of the maximum recommended month, where 30.5 days represented one month. Days spent underimmunized began to accumulate following the end of this 30.5-day grace period. For example, the first dose of diphtheria and tetanus toxoids and acellular pertussis combination vaccine (DTaP) is recommended at 2 months. A child who receives a valid DTaP by 91 days of age (i.e., (2 months* 30.5) + 30.5 day grace period = 91.5 days) is considered to have been vaccinated age-appropriately and does not accumulate any underimmunized time. By comparison, a child who receives their first DTaP at 94 days of age has accumulated 2 days of underimmunized time (i.e., underimmunized for days 92 and 93, and up-to-date on day 94 for the first dose of DTaP).

### Description of covariates

In addition to the PCV exposure cohorts, other vaccine policy and temporal factors related to immunization coverage were considered. Between July and September 1999, providers were encouraged to delay initiation of hepatitis B vaccination for low-risk infants from birth to 2–6 months of age because of safety concerns about thimerosal[12]. While resumption of hepatitis B birth vaccination practices was recommended after regulatory approval of the first thimerosal-free hepatitis B vaccine formulation in September 1999,[13] reinstatement of universal birth vaccination policies occurred slowly [14-17]. Two indicators were included in the regression models to account for potential disruptions in immunization coverage during the pre-PCV period, related the temporary hepatitis B birth dose suspension ("HB delay" cohort: date of birth between July – September 1999) and to incomplete resumption of hepatitis B birth vaccination practices ("HB carryover" cohort: date of birth between October 1999 – January 2000).

Temporal trends in immunization coverage were modeled using four variables based on birth month cohort: a linear slope was fit for the entire study period, and three additional linear trends were included to estimate changes in slope during the HB delay, HB carryover, and post-PCV periods relative to the pre-PCV baseline trend.

An indicator variable was included to account for a potential change in the level of immunization coverage after integration of PCV into routine practice ("PCV routine": July – November 2000) compared to the initial PCV adoption period ("PCV adoption": February – June 2000). Selection of the July 2000 birth cohort as the transition point after which PCV entered routine use was based on descriptive analyses of the adoption of PCV in the participating provider groups.

Finally, seasonal variation in immunization scheduling could affect the timeliness of vaccination and was entered into the time spent underimmunized regression models using indicator variables for calendar month of birth.

**Figure 1:**
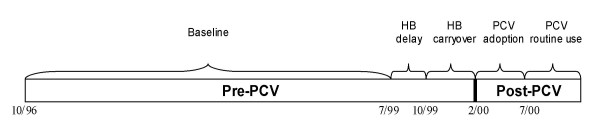
**Timing of PCV introduction and hepatitis B policy periods**. "Pre-PCV" and "Post-PCV" describe the timing of the exposure birth cohorts. The "pre-PCV" cohort includes children born prior to introduction of pneumococcal conjugate vaccine (date of birth between October 1996 – January 2000) while the "post-PCV" cohort includes children born after introduction of pneumococcal conjugate vaccine to the end of the study period (date of birth between February – November 2000). "HB delay" refers to children born during the temporary hepatitis B birth dose suspension (July – September 1999). "HB carryover" refers to children born after reinstatement of hepatitis B birth vaccination recommendations and before introduction of pneumococcal conjugate vaccine (October 1999 – January 2000). "PCV adoption" represents the first five months following introduction of pneumococcal conjugate vaccine and the period of initial uptake of pneumococcal conjugate vaccine in the four study sites (date of birth between February – June 2000). "PCV routine" represents the five-month period after adoption of pneumococcal conjugate vaccine had occurred (date of birth between July – November 2000).

Figure 1 illustrates how the hepatitis B and PCV policy variables are temporally related.

### Statistical analysis

Logistic regression was used to assess the association between PCV introduction and a child's probability of being up-to-date at 13 months.

For the second outcome, time spent underimmunized, we expected that the majority of children would be vaccinated age-appropriately (i.e., zero days spent underimmunized by 13 months), and a two-part modelling approach[18] was applied. First, a logistic regression model was used to assess the association between PCV introduction and a child's probability of having received all vaccines age-appropriately by 13 months of age. Then, among children who spent at least one day underimmunized, negative binomial regression was used to evaluate the impact of PCV introduction on the discrete outcome, number of days spent underimmunized by 13 months. Negative binomial regression accounts for overdispersion in the data[19] and provides relative rate estimates for the association between PCV introduction and the number of days spent underimmunized.

Analyses were stratified by provider group because differences in baseline immunization coverage, differing concern regarding multiple injections, and provider group-specific decisions to use combination vaccines may have differentially affected the impact of PCV introduction across the provider groups. To allow comparisons across sites, the set of variables included in the regression analysis for each outcome was fixed across the provider groups.

**Figure 2:**
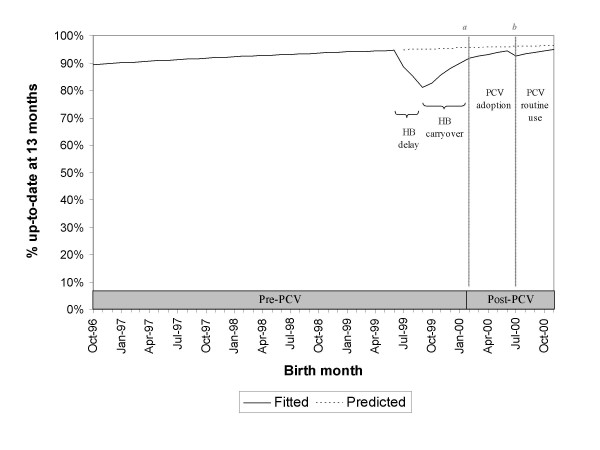
**PCV introduction and immunization coverage: illustration of primary contrasts**. Graph uses data from Group C as an example. ---: coverage based on multivariate regression models and the observed data. - - - -: coverage predicted from the pre-PCV baseline trend. ***Time point a***: February 2000 birth cohort, start of the PCV adoption period. ***Contrast a ***compares immunization coverage for the February 2000 birth cohort based on the observed data to that predicted from the pre-PCV baseline trend. ***Time point b***: July 2000 birth cohort, start of the PCV routine period. ***Contrast b ***compares immunization coverage for the July 2000 birth cohort based on the observed data to that predicted from the pre-PCV baseline trend.

Based on the regression model, we estimated the effect of introduction of PCV at two points – (1) immediately following PCV introduction (February 2000 birth cohort) and (2) after PCV was integrated into routine use (July 2000 birth cohort) – comparing each to the outcome as predicted from the pre-PCV baseline trend. Comparison of the PCV routine use period to the predicted baseline trend was considered of primary interest because it measures the impact of addition of PCV, allowing for a period of adjustment to the new PCV policy. Figure 2 illustrates the calculation of these two contrasts for the up-to-date outcome as an example. Given that the baseline proportion of children who were up-to-date or who were vaccinated age-appropriately was expected to be high, under these conditions, odds ratios should not be interpreted as approximately the relative risk. Absolute differences in coverage were also provided, comparing the probability fitted from multivariate regression models based on the observed data ("fitted probability") to the "predicted probability" extrapolated from the baseline trend (e.g., absolute difference_Feb2000 _= [fitted probability]_Feb2000 _- [predicted probability]_Feb2000_). All analyses were performed using SAS software, Version 8.2 of the SAS System for Windows (SAS Institute, Cary, NC).

## Results

### Study population and adoption of PCV

In the four provider groups, 38,588 children met study inclusion criteria. Due to the identification of a potential disruption in the immunization tracking system during the early part of the study period in one of the provider groups, the study population for that site (Group D) was additionally restricted to children born between November 1998 and November 2000. This resulted in a final study population of 33,319 children.

Following its introduction in February 2000, the rate of adoption of PCV varied but was relatively rapid across the four sites. Among children born in July 2000, over 85% of children in each of three provider groups (Groups A, B, and D) received PCV at their 2-month visit. In Group C, while only 24% of the July 2000 birth cohort had received PCV at a 2-month visit, 76% in the August 2000 cohort had done so. Notwithstanding the age at which the PCV series was initiated, between 92% (Group C) and 96% (Group D) of the July 2000 birth cohort had received three shots of PCV by 13 months.

### Impact of PCV recommendations on probability of being up-to-date at 13 months

**Figure 3:**
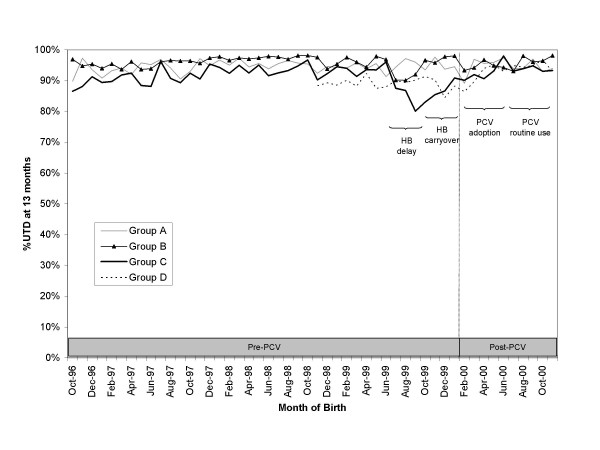
**Percent up-to-date for non-PCV recommended vaccines at 13 months**. Children were grouped by the month and year of their birth. Note: Data between April – October 1998 were considered incomplete in Group D due to a potential disruption in their immunization information system during the pre-PCV baseline period.

Overall, 93% of children were up-to-date at age 13 months. In each provider group, the percent of 13-month-olds who were up-to-date either was maintained or increased slightly from the pre-PCV to the post-PCV cohorts (Figure 3).

In Group C, children born at the start of the PCV adoption period were less likely to be up-to-date at 13 months compared to the pre-PCV baseline after multivariate adjustment for the HB delay and carryover periods (OR = 0.5, 95% CI: 0.4 – 0.8) (Table 2). This decrease persisted even after PCV entered routine use (Group C: OR = 0.5, 95% CI: 0.3 – 0.8), representing 3% fewer children in Group C who were up-to-date compared to the predicted baseline trend. Additional analyses indicated that children in Group C were less likely to be up-to-date in the HB delay (OR = 0.4; 95% CI: 0.3 – 0.6) and HB carryover (OR = 0.25; 95% CI: 0.17 – 0.35) periods compared to baseline, preceding the introduction of PCV. This suggests that the decrease observed in post-PCV period in Group C may have been due to the lingering effects of the hepatitis B birth dose suspension. In contrast, PCV introduction was not significantly associated with a child's probability of being up-to-date at 13 months in the three other provider groups.

**Table 2:** PCV introduction and probability of being up-to-date at 13 months

	***Contrast a***: PCV adoption vs. predicted baseline				***Contrast b***: PCV routine vs. predicted baseline			
Group	Odds ratio	95% CI	Fitted probability	Predicted probability	Odds ratio	95% CI	Fitted probability	Predicted probability

Group A	1.0	0.5 – 1.8	0.95	0.95	0.8	0.4 – 1.4	0.94	0.95
Group B	0.5	0.2 – 1.2	0.96	0.98	0.7	0.3 – 1.5	0.97	0.98
Group C	0.5	0.4 – 0.8	0.92	0.95	0.5	0.3 – 0.8	0.93	0.96
Group D	1.2	0.5 – 2.9	0.90	0.89	2.0	0.6 – 7.0	0.94	0.88

***Contrast a ***compares a child's probability of being up-to-date at 13 months among the February 2000 birth cohort to that predicted from the pre-PCV baseline trend. ***Contrast b ***compares a child's probability of being up-to-date at 13 months among the July 2000 birth cohort to that predicted from the pre-PCV baseline trend. For each contrast, the "fitted probability" was the probability of being up-to-date as fitted from the multivariate regression models based on the observed data and the "predicted probability" was extrapolated from the pre-PCV baseline trend.

### Impact of PCV recommendations on time spent underimmunized by 13 months

Overall, 70% of children received all non-PCV recommended vaccinations without incurring any underimmunized time. Among the 30% of children who were ever delayed for at least one vaccination, a median of 122 days (interquartile range: 33 – 212) was spent underimmunized during the first 13 months of life.

Multivariate-adjusted results are provided only for Groups A, B, and C because the truncated study period for Group D limited our ability to fit the full multivariate model for this population (Table 3). Children born at the start of the PCV adoption period were less likely to receive all vaccinations without delay in Group B (OR = 0.4, 95% CI: 0.3–0.5) and in Group C (OR = 0.33, 95% CI: 0.26 – 0.42). These differences persisted through the start of the PCV routine period (Group B OR = 0.4, 95% CI: 0.3 – 0.6; Group C OR = 0.5, 95% CI: 0.4 – 0.7), representing 16% (Group B) and 14% (Group C) fewer children among the July 2000 birth cohort who spent no time underimmunized compared to baseline. In Group A, no difference in timeliness of immunization delivery was noted following PCV introduction.

**Table 3:** PCV introduction and probability of never being underimmunized by 13 months of age

	***Contrast a***: PCV adoption vs. pre-PCV baseline				***Contrast b***: PCV routine vs. pre-PCV baseline			
Group	Odds ratio	95% CI	Fitted probability	Predicted probability	Odds ratio	95% CI	Fitted probability	Predicted probability

Group A	1.1	0.7 – 1.6	0.83	0.82	1.1.	0.7 – 1.8	0.86	0.85
Group B	0.4	0.3 – 0.5	0.55	0.77	0.4	0.3 – 0.6	0.65	0.81
Group C	0.33	0.26 – 0.42	0.34	0.60	0.5	0.4 – 0.7	0.58	0.72

***Contrast a ***compares a child's probability of spending zero days underimmunized by 13 months among the February 2000 birth cohort based on the observed data to that predicted from the pre-PCV baseline trend. ***Contrast b ***compares a child's probability of spending zero days underimmunized by 13 months among the July 2000 birth cohort based on the observed data to that predicted from the pre-PCV baseline trend. For each contrast, the "fitted probability" was the probability of spending no time underimmunized as fitted from the multivariate regression models and the "predicted probability" was extrapolated from the pre-PCV baseline trend.

Among children ever-delayed, children born during the PCV adoption period in Group C spent 1.5 times as many days underimmunized (CI: 1.3 – 1.8) compared to the predicted baseline trend. This increase in days spent underimmunized persisted among the PCV routine use cohort compared to the baseline trend (RR: 1.4, 95% CI: 1.2 – 1.8), representing a delay of 103 days versus 73 days on average, among children in Group C who were ever-underimmunized. In contrast, no significant difference in the number of days spent underimmunized was found in the other two Groups, for either the PCV adoption cohort (Group A RR: 1.1, 95% CI: 0.7–1.7; Group B RR: 1.1, 95% CI: 0.8–1.4) or the PCV routine use cohort (Group A RR: 1.4, 95% CI: 0.8–2.2; Group B RR: 0.9; 95% CI: 0.6–1.2), compared to the baseline trend.

### Introduction of PCV and immunization coverage for individual vaccine series

Additional analyses suggest that the decrease in probability of being up-to-date observed in Group C was not driven by disruptions in one vaccine series alone. Children in Group C were less likely to be up-to-date at the start of the PCV adoption period for hepatitis B vaccination (OR = 0.4, 95% CI: 0.2 – 0.8) as well as for each of the other vaccine series (polio: OR = 0.48, 95% CI: 0.24 – 0.96; DTP: OR = 0.65, 95% CI: 0.41 – 1.03; Hib: OR = 0.60; 95% CI: 0.38 – 0.96). These effects persisted after PCV entered routine use for the polio (OR = 0.48, 95% CI: 0.23–1.01), DTP (OR = 0.5, 95% CI: 0.3–0.9) and Hib (OR = 0.5, 95% CI: 0.3–0.9) vaccine series compared to baseline. In the three provider groups where PCV introduction was not associated with changes in up-to-date coverage among 13-month-olds, no systematic differences in coverage for individual vaccine series were observed. Similar broader disruptions across individual vaccine series were observed for the impact of PCV on the time spent underimmunized by13 months (data not shown).

## Discussion

In our study of four large provider group populations, the introduction of PCV was not associated with a substantial adverse impact on a child's probability of being up-to-date at 13 months for non-PCV recommended childhood vaccines. We did find moderate increases in time spent underimmunized in some provider groups, although these delays likely did not result in clinical harm. Our findings suggest that the timeliness of immunization for several vaccine series was likely adversely affected by either PCV introduction or the hepatitis B birth dose suspension, although it was not possible to disentangle the effects of these two policy changes. Overall, our findings support continued vigilance during changes in immunization policy in order to mitigate unintended delays in vaccine delivery that may arise due to concerns about multiple injections.

Our finding of no major impact of PCV introduction on up-to-date status is consistent with reports that increases in the number of vaccine injections have not led to clinically important reductions in immunization coverage[11]. While providers and parents have expressed concerns regarding multiple injections,[4,6,20,21] many parents still prefer that all recommended vaccines be given at one visit[20]. Recent surveys also suggest that providers are now more willing to administer multiple injections[7,21,22]. These findings are consistent with results from the 2003 National Immunization Survey,[23] which indicate that coverage levels for other childhood vaccines among children aged 19–35 months of age had been maintained during the adoption of PCV.

The heterogeneity in the response to PCV introduction observed across the sites may have arisen due to a variety of reasons. Responses to hepatitis B policy changes differed across the provider groups, and only those sites that experienced disruptions during the hepatitis B delay and carryover periods had lower immunization coverage following PCV introduction. If the effects of the hepatitis B vaccination policy reversals lingered as PCV entered routine use in our health plan populations (i.e., 10 months after reinstatement of the birth vaccination recommendations), our study would be unable to fully disentangle the relative contributions of these two policies. Availability of the hepatitis B-Hib combination vaccine may simplify immunization scheduling by reducing the total number of shots a child receives during the first year of life,[24] and the differential adoption of the combination hepatitis B-hib vaccine may have influenced the variation observed across provider groups. The routine use of the combination hepatitis B-Hib vaccine in Group A throughout the study period may have made immunization scheduling less sensitive to the temporary hepatitis B birth dose suspension or alleviated concerns about multiple injections related to the introduction of PCV.

Implementation of the hepatitis B-Hib combination vaccine during the study period in Group C and Group D may also have influenced the impact of PCV introduction on immunization coverage in these settings; however, the coincident timing of the implementation of the hepatitis B-Hib combination vaccine with the temporary hepatitis B birth dose suspension (Group D) and PCV adoption (Group D) limits our ability to determine separate effects for these policy changes in our analysis. We also compared immunization coverage for non-PCV vaccinations following PCV introduction with predictions of immunization coverage extrapolated from baseline trends. Dependent on the level of baseline immunization coverage, expectation of a continued constant trend in immunization coverage may have yielded an overestimate of the extent of disruption in immunization coverage in some settings. It is reassuring that PCV introduction was not associated with clinically important reductions in immunization coverage, even given expectations of a linear trend predicted from baseline. Finally, flexibility in immunization guidelines or expectations regarding the simultaneous administration of vaccines may vary across sites and influence variation in immunization scheduling.

This study evaluates the impact of introduction of the PCV policy and not whether administration of PCV to a given child affects that individual's probability of being up-to-date. Given the rapid adoption of PCV in the four provider groups, we are able to estimate the net effect of integration of PCV into the childhood immunization schedule. Information on certain demographic factors (e.g., race/ethnicity, socioeconomic status) was not routinely collected across these settings, and we were unable to directly assess the effects of these characteristics in the analysis. However, by comparing birth cohorts across the study period, only those characteristics whose distribution in the study population changed concurrently with the time of introduction of PCV can act as potential confounders. No major changes in the coverage plans offered by these health plans occurred at the time of PCV introduction, and we therefore would not expect the demographic characteristics of the enrolled populations in these provider groups to have changed dramatically concurrent with introduction of the new PCV policy.

Finally, this study was conducted in health plan populations, who are mostly privately insured and have good access to health care. The study population was additionally restricted to children who were continuously enrolled during the first year of life; these children are likely to have experienced less scattering of their immunization records and to have had more opportunities to catch-up on immunizations. While children in the study population are thus likely to have higher overall immunization coverage than might be expected for the general population, comparison across similarly restricted birth cohorts remains valid to evaluate the potential impact of the introduction of PCV. In addition, earlier evaluations of the impact of the transition from oral polio vaccine to inactivated (injected) polio vaccine have yielded similar findings of a lack of an adverse effect on immunization coverage among children enrolled in managed care populations[11] and among children receiving vaccinations in public clinics[20].

## Conclusion

The continued development and addition of new vaccines to the childhood immunization schedule is likely to exacerbate concerns regarding simultaneous multiple injections. Our findings indicate that these provider groups remained capable of absorbing the most recent increases in multiple injections with minimal impact on up-to-date measures. However, the timeliness of delivery may have been affected by the introduction of PCV and the suspension of the birth dose of hepatitis B vaccine. This study highlights the continuing need to monitor the impact of new vaccines as well as vaccine-safety related policy decisions that affect immunization scheduling.

## Competing interests

The author(s) declare that they have no competing interests.

## Authors' contributions

NDL participated in the design of the study, analysis and interpretation of results, and manuscript preparation and revision. KK participated in the design of the study, interpretation of results, and manuscript revision. KAC participated in the design of the study, interpretation of results, and manuscript revision. XY participated in the acquisition of data, analysis and interpretation of the results, and manuscript preparation. EKF, SX, FW, and JM were involved in the acquisition of data, interpretation of results, and manuscript revision. JS was involved in the interpretation of results and manuscript revision. TAL participated in the design of the study, acquisition of data, analysis and interpretation or results, and manuscript revision.

## Pre-publication history

The pre-publication history for this paper can be accessed here:


